# Gemcitabine-based *versus *fluoropyrimidine-based chemotherapy with or without platinum in unresectable biliary tract cancer: a retrospective study

**DOI:** 10.1186/1471-2407-8-374

**Published:** 2008-12-18

**Authors:** Mi-Jung Kim, Do-Youn Oh, Se-Hoon Lee, Dong-Wan Kim, Seock-Ah Im, Tae-You Kim, Dae Seog Heo, Yung-Jue Bang

**Affiliations:** 1 Department of Internal Medicine, Seoul National University Hospital, Seoul National University College of Medicine, Seoul, Korea; 2 Cancer Research Institute, Seoul National University College of Medicine, Seoul, Korea

## Abstract

**Background:**

There is no standard palliative chemotherapy regimen in biliary tract cancers (BTC). Fluoropyrimidine or gemcitabine, with or without platinum, are most frequently used. We conducted this study to clarify the efficacy of palliative chemotherapy in BTC.

**Methods:**

Patients with unresectable BTC treated with palliative chemotherapy between Oct 2001 and Aug 2006 at Seoul National University Hospital were reviewed retrospectively. Histologically confirmed cases of intrahepatic cholangiocarcinoma, gallbladder cancer, extrahepatic bile duct cancer, and ampulla of Vater carcinoma were enrolled. We analyzed the efficacy of regimens: gemcitabine (G) *versus *fluoropyrimidine (F) and with or without platinum (P).

**Results:**

A total of 243 patients were enrolled. 159 patients (65%) were male and the median age of the patients was 60 years (range 26–81). Intrahepatic cholangiocarcinoma, gallbladder cancer, extrahepatic bile duct cancer, and ampulla of Vater carcinoma were 92, 72, 58, and 21 cases, respectively. The median progression free survival (PFS) was 4.3 months (95% CI, 3.7–4.9) and median overall survival (OS) was 8.7 months (95% CI, 7.4–10.0). Ninety-nine patients received G-based chemotherapy (94 GP, 5 G alone), and 144 patients received F-based chemotherapy (83 FP, 61 F alone). The response rate (RR), disease control rate (DCR), PFS and OS of G-based chemotherapy *versus *F-based chemotherapy were 16.7% *vs*. 19.5% (P = 0.591), 52.8% *vs*. 58.9% (P = 0.372), 4.0 months *vs*. 4.3 months (P = 0.816), and 7.8 months *vs*. 9.1 months (P = 0.848), respectively. Sixty-six patients received F or G without P, and 177 patients received F or G with P. The RR, DCR, PFS and OS of chemotherapy without P *versus *chemotherapy including P were 12.7% *vs*. 20.6% (P = 0.169), 46.0% *vs*. 60.6% (P = 0.049), 3.3 months *vs*. 4.4 months (P = 0.887), and 10.6 months *vs*. 8.1 months (P = 0.257), respectively.

**Conclusion:**

In unresectable BTC, F-based and G-based chemotherapy showed similar efficacy in terms of RR, DCR, PFS and OS. The benefit of adding P to F or G was not significant except for DCR. Further prospective studies which define the efficacy of various chemotherapeutic regimens in BTC are warranted.

## Background

Biliary tract cancers (BTC) are relatively rare tumors with a poor prognosis. BTC account for less than 2% of all malignancies in the West [1], but BTC are more common in Korea and Japan accounting for approximately 4% of malignancies [2]. BTC are classified by locations, as intrahepatic cholangiocarcinoma, extrahepatic bile duct cancer, gallbladder carcinoma and ampulla of Vater carcinoma [3]. The only curative treatment is surgical resection, but over 75% of patients are unresectable, typically due to advanced stage at diagnosis [4]. Patients with unresectable BTC can be considered for palliative chemotherapy, which is reported to improve overall survival and quality of life over best supportive care [5], but a standard chemotherapy for BTC has not been established.

Most BTC studies contain only a small number of patients because of the low incidence of BTC, and the most frequently used and studied drug in BTC is 5-fluorouracil (5-FU), which is used alone or in combination with other agents and has a response rate of 0–30% [6-9]. Gemcitabine (G) was introduced in the treatment of BTC and has been reported to show a response rate of 10–30% [10-16]. G is being used alone or in combination with other agents, such as platinum and fluoropyrimidine [10-16]. Platinum (P), especially, has been reported to have single agent activity with a response rate of about 20% [17]. In addition, regimens that contain newer fluoropyrimidines (F), including capecitabine and S-1, have been reported to have response rates of 20–40% [18-21].

F and G have been two major drugs in this way in the treatment of unresectable BTC, but no superiority between F-based and G-based chemotherapy has been identified. The National Comprehensive Cancer Network (NCCN) guideline recommends 5-FU-based or G as first-line regimen in unresectable BTC. However, no studies directly comparing G with F in single or combination regimens have been undertaken. As the incidence of BTC in Korea is relatively higher than in the West, we have access to a relatively large BTC patient pool as a single institute. We conducted this study to demonstrate the efficacy of palliative chemotherapy in BTC and to find out the clue to the preferred regimen in a large BTC population. We particularly analyzed the efficacy of regimens: G vs. F and with P vs. without P.

## Methods

### Study Design

We retrospectively reviewed 258 consecutive patients with unresectable BTC who were treated with palliative chemotherapy between Oct 2001 and Aug 2006 at Seoul National University Hospital. Patients with adenocarcinomas arising from intrahepatic cholangiocarcinoma, gallbladder carcinoma, extrahepatic bile duct cancer, and ampulla of Vater carcinoma, which were histologically confirmed, were included.

Fluoropyrimidines used in this study included 5-FU, S-1, capecitabine and uracil-tegafur (UFT). 5-FU monotherapy consisted of 5-FU 375–500 mg/m^2^/day by bolus infusion alone or modulated with levofolinic acid (leucovorin) 25 mg/m^2^/day from day 1 to 5 every 4 weeks (Mayo regimen). S-1 was used alone at a dose of 40 mg/m^2 ^twice daily for 28 days, followed by a 14-day rest period, and capecitabine was used alone at a dose of 2500 mg/m^2^/day for 14 days, followed by a 7-day day rest period. UFT was used alone at a dose of 300 mg/m^2^/day for 28 days, followed by a 7-day rest period. 5-FU combination with P consisted of 5-FU administered intravenously at a dose of 1200 mg/m^2^/day over 10 hours from day 1 to 4 and cisplatin 60 mg/m^2^/day administered intravenously over 15 minutes on day 1 every 3 weeks. S-1 or capecitabine was also administered with a same dosage and schedule of cisplatin. Gemcitabine monotherapy consisted of gemcitabine 1000 mg/m^2^/day over 30 minutes on day 1, 8, and 15 every 4 weeks. In addition, G combination with P consisted of gemcitabine administered intravenously at a dose of 1200 mg/m^2^/day over 30 minutes on day 1 and 8 and cisplatin administered intravenously at a dose of 60 mg/m^2^/day over 15 minutes on day 1 every 3 weeks. Platinum compounds included cisplatin, and oxaliplatin. When oxaliplatin was used instead of cisplatin, it was used at a dose of 130 mg/m^2^/day over 2 hours with S-1 or capecitabine on day 1 every 3 weeks or 100 mg/m^2^/day over 2 hours with gemcitabine on day 1 every 2 weeks.

We excluded 14 patients who received both G and F simultaneously as first-line chemotherapy and one patient who received a regimen other than G or F (paclitaxel and cisplatin) because they were inconsistent with the aim of this study. In total, 243 patients were enrolled finally in this study. We assessed response to chemotherapy in each patient according to the RECIST (Response Evaluation Criteria in Solid Tumors) [22]. Appropriate imaging studies, including CT scans, were usually performed every two cycles. The proportions of patients in whom biliary drainage procedures, such as biliary stent and percutaneous transhepatic biliary drainage (PTBD), were performed were evaluated, and the infections which frequently occurred to patients with BTC, such as cholangitis and liver abscess, were defined as disease-associated infections. Our study was approved by the institutional review board of the Seoul National University Hospital, Seoul, Korea.

### Statistical Analysis

We analyzed the efficacy of first-line chemotherapy. Specifically, we compared the efficacy of G-based vs. F-based regimens, and with or without P. The median overall survival (OS) and median progression free survival (PFS) were measured. OS was calculated from the first day of palliative chemotherapy to the day of death or last followed-up. PFS was calculated from the first day of palliative chemotherapy to the day of progression or last followed-up. Descriptive statistics were reported as medians and proportions, and χ^2 ^test was used to compare baseline characteristics between chemotherapy groups. Confidence intervals (95% CI) for response rate and disease control rate were estimated by binomial distribution [23]. Survival curves were estimated by the Kaplan-Meier method for OS and PFS, and 95% CI for the median time to event was calculated by Greenwood's formula [23]. The log-rank test was used to compare the distribution of survival between groups. Statistical analyses were performed by the statistical software package SPSS version 12.0 K (SPSS Inc. Chicago, IL, USA). A probability value of 0.05 indicated statistical significance.

## Results

### Patient Characteristics

We finally enrolled 243 patients with unresectable BTC (Table 1). There were 159 (65%) males and 84 (35%) females. The median age of the patients was 60 years (range 26–81 years). The median follow-up duration was 6.5 months (range 0.0–47.8 months). ECOG performance status (PS) at baseline was either 0 or 1 in 215 patients (88%). There were 36 patients (15%) who underwent palliative surgery. Intrahepatic cholangiocarcinoma, gallbladder carcinoma, extrahepatic bile duct cancer, and ampulla of Vater carcinoma were 92 (38%), 72 (30%), 58 (24%), and 21 (9%) cases, respectively. Sixty-four patients (26%) had biliary stents or bypasses for obstructive disease during treatment. Disease-associated infections were detected in 62 patients (26%).

**Table 1:** Patient characteristics

**Characteristics**	**No. of patients**	**%**
Total Number	243	
Median age, years (range)	60 (26–81)	
Sex		
Male	159	65
Female	84	35
ECOG performance status at baseline		
0 – 1	215	88
2	28	12
Disease		
Intrahepatic cholangiocarcinoma	92	38
Gallbladder cancer	72	30
Extrahepatic bile duct cancer	58	24
Ampulla of Vater carcinoma	21	9
Recurrent	84	35
Initially metastatic	154	63
Biliary stent or bypass during treatment	64	26
Disease-associated infection during treatment (cholangitis, liver abscess)	62	26

ECOG, Eastern Cooperative Oncology Group

In a total, 144 patients (59%) received F-based chemotherapy. They took oral F such as capecitabine, S1, and UFT besides intravenous 5-FU. The proportions of the patients who took 5-FU, S1, capecitabine, and UFT were 44%, 39%, 15% and 2%, respectively. The proportions of the patients who were treated in combination with P among these patients were 27%, 89%, 73% and 0%, respectively (Table 2). Ninety-nine patients (41%) received G-based chemotherapy. Most (95%) of them were treated in combination with P. One hundred and seventy-seven (73%) patients received chemotherapy combined with P. Most of the patients received cisplatin, and only 9 patients (5%) received oxaliplatin.

**Table 2:** Number of patients according to the regimens

Type of Regimens	With P	Without P	Total
			
	N (%)	N (%)	N (%)
F-based regimens	83 (58)	61 (42)	144 (100)
5-FU	17	46	63
S1	50	6	56
Capecitabine	16	6	22
UFT	0	3	3
G-based regimens	94 (95)	5 (5)	99 (100)

Total	177 (73)	66 (27)	243 (100)

P, platinum; F, fluoropyrimidines; 5-FU, 5-fluorouracil; UFT, uracil-tegafur; G, gemcitabine

The median age, sexual distribution, baseline PS, and the distribution of disease types were not significantly different between F-based and G-based chemotherapy groups (Table 3). There was a significant difference in biliary stents or bypasses between the groups (P = 0.040), but no significant difference in disease-associated infections, which are considered to develop easily under these conditions.

**Table 3:** Comparison of F-based group *vs*. G-based group

Characteristics	F Group (%)	G Group (%)	*P *value
Total Number	144	99	
Median age, years (range)	60 (range 29–80)	61 (range 26–81)	
Sex			0.831
Male	95 (66.0)	64 (64.6)	
Female	49 (34.0)	35 (35.4)	
ECOG performance status at baseline			0.06
0 – 1	132 (91.7)	83 (83.8)	
≥ 2	12 (8.3)	16 (16.2)	
Disease			0.966
Intrahepatic cholangiocarcinoma	54 (37.5)	38 (38.4)	
Gallbladder cancer	42 (29.2)	30 (30.3)	
Extrahepatic bile duct cancer	36 (25.0)	22 (22.2)	
Ampulla of Vater carcinoma	12 (8.3)	9 (9.1)	
Relapsed disease	51 (35.4)	33 (33.3)	0.737
Biliary stent or bypass during treatment	31 (21.5)	33 (33.3)	0.04
Disease associated infection during treatment (cholangitis, liver abscess)	31 (21.5)	31 (31.3)	0.086
Duration from off chemotherapy to death	3.5 months (95% CI, 2.9–4.0)	3.3 months (95% CI, 2.2–4.3)	0.752

ECOG, Eastern Cooperative Oncology Group; F, fluoropyrimidines; G, gemcitabine; CI, confidence interval

When the patients were divided according to the presence of platinum compounds, age and baseline PS in the two groups were significantly different (Table 4). Other characteristics showed no difference between groups. In our study, elderly patients tended to be treated with oral chemotherapeutic agents, such as S-1, capecitabine and UFT, which all belonged to non-platinum group. This fact seems to contribute to more increased median age in non-platinum group. Really, patients treated with oral chemotherapeutic agents were significantly older than those treated with intravenous chemotherapeutic agents (65 years *vs*. 59 years, P = 0.025).

**Table 4:** Comparison of platinum group *vs*. non – platinum group

Characteristics	Platinum Group (%)	Non-Platinum Group (%)	*P *value
Total Number	177	66	
Median age, years (range)	58 (range 26–81)	64 (range 29–80)	0.003
Sex			0.247
Male	112 (63.3)	47 (71.2)	
Female	65 (36.7)	19 (28.8)	
ECOG performance status at baseline			0.047
0 – 1	161 (91.0)	54 (81.8)	
≥ 2	16 (9.0)	12 (18.2)	
Disease			0.361
Intrahepatic cholangiocarcinoma	72 (40.7)	20 (30.3)	
Gallbladder cancer	51 (28.8)	20 (30.3)	
Extrahepatic bile duct cancer	38 (21.5)	22 (22.2)	
Ampulla of Vater carcinoma	16 (9.0)	5 (7.6)	
Relapsed disease	62 (35.0)	22 (33.3)	0.805
Biliary stent or bypass during treatment	46 (26.0)	18 (27.3)	0.84
Disease associated infection during treatment (cholangitis, liver abscess)	43 (24.3)	19 (28.8)	0.475
Duration from off chemotherapy to death	3.3 months (95% CI, 2.6–3.9)	4.0 months (95% CI, 2.6–5.5)	0.071

ECOG, Eastern Cooperative Oncology Group; CI, confidence interval

### Treatment Outcomes

The overall response rate (RR) of first-line chemotherapy was 18.3% (95% CI, 13.2–23.5%) and the disease control rate (DCR) was 56.4% (95% CI, 49.8–63.0%). The median duration of first-line chemotherapy was 2.1 months (range, 0.0–13.2 months), and median duration of response was 4.5 months (range, 1.1–13.1 months). The median OS was 8.7 months (95% CI, 7.4–10.0 months) and median PFS was 4.3 months (95% CI, 3.7–4.9 months).

Treatment outcomes including survival time were also analyzed according to disease types. The median OS was 7.6 months (95% CI, 6.0–9.1 months) for intrahepatic cholangiocarcinoma, 8.8 months (95% CI, 6.4–11.1 months) for gallbladder carcinoma, 9.4 months (95% CI, 6.0–12.7 months) for extrahepatic bile duct cancer, and 11.7 months (95% CI, 8.2–15.3 months) for ampulla of Vater carcinoma. The median PFS was 4.3 months (95% CI, 3.3–5.3 months) for intrahepatic cholangiocarcinoma, 4.3 months (95% CI, 3.3 – 5.3 months) for gallbladder carcinoma, 4.4 months (95% CI, 2.7–6.1 months) for extrahepatic bile duct cancer, and 2.9 months (95% CI, 1.4–4.4 months) for ampulla of Vater carcinoma. There was no statistically significant difference in either OS or PFS among disease types. RR and DCR also showed no statistically significant differences among disease types. These results were the same in subgroup analysis of gallbladder carcinoma *vs*. all other BTC. Gallbladder carcinoma showed similar efficacy to all other BTC in all aspects of RR, DCR, OS, and PFS (RR 20.6% *vs*. 16.8%, P = 0.494; DCR 63.5% *vs*. 53.3%, P = 0.165; OS 8.8 months (95% CI, 6.7–10.8 months) *vs*. 8.1 months (95% CI, 7.0–9.2 months), P = 0.370; PFS 4.3 months (95% CI, 3.4–5.2 months) *vs*. 4.0 months (95% CI, 3.3–4.7 months), P = 0.515). When disease types were classified into two groups, i.e. intrahepatic and extrahepatic bile duct cancers, the extrahepatic bile duct cancer group had significantly longer median OS than the intrahepatic bile duct cancer group (9.9 months *vs*. 7.6 months, P = 0.025).

#### Gemcitabine vs. fluoropyrimidines

**Figure 1:**
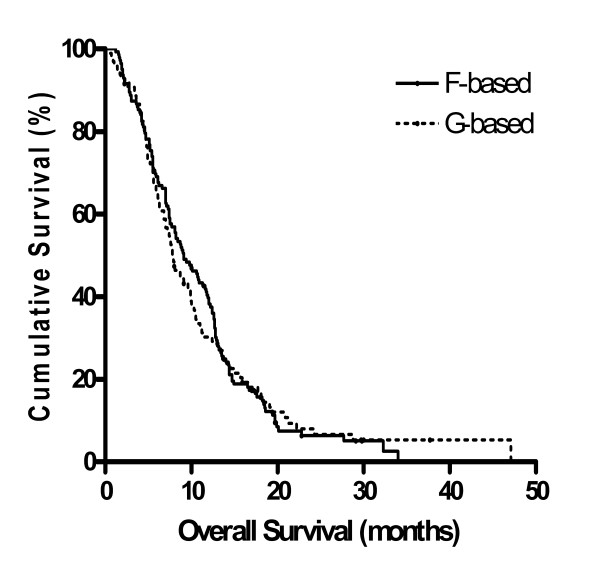
**Overall survival of F-based *vs*. G-based chemotherapy estimated by the Kaplan-Meier method; F: fluoropyrimidines; G: gemcitabine**.

Ninety-nine patients received G-based chemotherapy with or without P (94 GP, 5 G alone) as first-line chemotherapy, while 144 patients received F-based chemotherapy with or without P as first-line chemotherapy (83 FP, 61 F alone) (Table 5). The RR and DCR of G-based *vs*. F-based chemotherapy were 16.7% *vs*. 19.5% (P = 0.591) and 52.8% *vs*. 58.9% (P = 0.372), respectively. The median PFS and OS of G-based *vs*. F-based chemotherapy were 4.0 months *vs*. 4.3 months (P = 0.816), and 7.8 months *vs*. 9.1 months (P = 0.848), respectively (Figure 1). F-based regimens showed better RR and DCR than G-based regimens, but showed no statistically significant difference. Similarly, the median PFS and OS were better in F-based regimens, but not significantly different.

**Table 5:** Treatment outcomes of fluoropyrimidines *vs*. gemcitabine

	F based	G based	*P *value
Number of patients	144	99	< 0.0001
With P	83	94	
Without P	61	5	
RR (%)	19.5	16.7	0.591
DCR (%)	58.9	52.8	0.372
PFS (months)	4.3 (95% CI, 3.3–5.3)	4.0 (95% CI, 2.9–5.1)	0.816
OS (months)	9.1 (95% CI, 7.1–11.1)	7.8 (95% CI, 6.5–9.2)	0.848

F, fluoropyrimidines; G, gemcitabine; P, platinum; RR, response rate; DCR, disease control rate; PFS, progression free survival; CI, confidence interval; OS, overall survival

#### Platinum-containing regimen vs. platinum non-containing regimen

**Figure 2:**
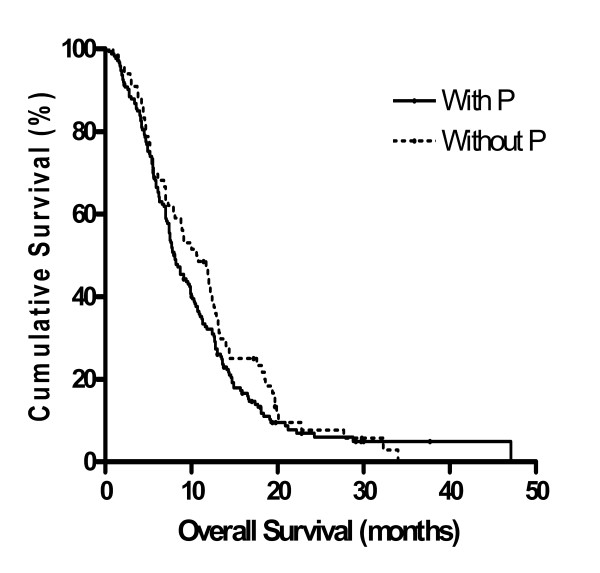
**Overall survival of chemotherapy with P *vs*. chemotherapy without P estimated by the Kaplan-Meier method; P: platinum**.

Sixty-six patients received G or F without P, and 177 patients received G or F with P (Table 6). The RR and DCR of chemotherapy without P *vs*. chemotherapy including P were 12.7% *vs*. 20.6% (P = 0.169) and 46.0% *vs*. 60.6% (P = 0.049), respectively. The median PFS and OS of chemotherapy without P *vs*. chemotherapy including P were 3.3 months *vs*. 4.4 months (P = 0.887), and 10.6 months *vs*. 8.1 months (P = 0.257) (Figure 2), respectively. The addition of P to G-based or F-based regimens caused statistically significant increase in DCR, but the benefit of adding P was minimal in the RR, OS, and PFS. These results were the same in a subgroup of patients less than 65 years. The RR, DCR, PFS, and OS of chemotherapy without P *vs*. chemotherapy including P in the subgroup less than 65 years were 12.1% *vs*. 21.7% (P = 0.225), 33.3% *vs*. 61.9% (P = 0.004), 2.4 *vs*. 4.4 months (P = 0.387), and 9.0 *vs*. 9.6 months, (P = 0.540), respectively.

**Table 6:** Treatment outcomes of with platinum *vs*. without platinum

	With Platinum	Without Platinum	*P *value
Number of patients	177	66	
RR (%)	20.6	12.7	0.169
DCR (%)	60.6	46.0	0.049
PFS (months)	4.4 (95% CI, 3.7–5.1)	3.3 (95% CI, 2.3–4.3)	0.887
OS (months)	8.1 (95% CI, 7.0–9.3)	10.6 (95% CI, 7.9–13.2)	0.257

RR, response rate; DCR, disease control rate; PFS, progression free survival; CI, confidence interval; OS, overall survival

#### Gemcitabine with platinum vs. fluoropyrimidine with platinum

Ninety-four patients received G with P, and 83 patients received F with P (Table 7). The RR, DCR, PFS, and OS of GP *vs*. FP were 17.6% *vs*. 24.3%, (P = 0.310), 54.8% *vs*. 67.6%, (P = 0.103), 4.3 months *vs*. 4.6 months, (P = 0.787), and 8.0 months *vs*. 8.1 months, (P = 0.357), respectively. Patients in G-based group (94 GP, 5 G alone) more frequently received combination chemotherapy with P compared with those in F-based group (83 FP, 61 F alone) (P < 0.0001), but the efficacy of GP was not significantly different from that of FP in all aspects.

**Table 7:** Treatment outcomes of FP *vs*. GP

	FP	GP	*P *value
Number of patients	83	94	
RR (%)	24.3	17.6	0.310
DCR (%)	67.6	54.8	0.103
PFS (months)	4.6 (95% CI, 3.5–5.7)	4.3 (95% CI, 3.6–5.0)	0.787
OS (months)	8.1 (95% CI, 7.0–9.3)	8.0 (95% CI, 6.0–10.0)	0.357

F, fluoropyrimidines; P, platinum; G, gemcitabine; RR, response rate; DCR, disease control rate; PFS, progression free survival; CI, confidence interval; OS, overall survival

#### Post-progression treatment

In a total of 243 patients, 129 (53%) received second-line chemotherapy after progression in first-line chemotherapy. Among 144 F-pretreated patients, 76 received second-line F-based or G-based chemotherapy, while 41 among 99 G-pretreated patients received second-line F-based chemotherapy. In F-pretreated group, there were 52 in G-based chemotherapy group and 24 in F-reused group. On the other hand, no patients in G-pretreated group reused G-based chemotherapy.

## Discussion

There is no defined standard palliative chemotherapy regimen for unresectable BTC. The goal of our study was to demonstrate the impact of palliative chemotherapy and to compare the efficacy of the most frequently used agents in BTC. Our results indicate that F-based regimens have a trend to better RR and DCR, and longer median PFS and OS than G-based regimens, but the difference failed to show statistical significance. The efficacy of F-based regimens, alone or in combination with other drugs, was analyzed, and the RR and DCR of F-based regimens were 19.5% and 58.9%, respectively. These results were comparable to those reported in other studies [9]. Ducreux *et al*. showed that a combination regimen of 5-FU, folinic acid and cisplatin had a 19% RR and a 44% DCR. The median PFS of 3.3 months and OS of 8.0 months in their study were similar to the results in our study, 4.3 months and 9.1 months. Oral F, such as capecitabine, S-1 and UFT, have also been evaluated in BTC. These agents, alone or in combination with cisplatin, doxorubicin, and epirubicin, showed significant antitumor activity [18-21,24]. In the present study, 56% of patients who received F-based chemotherapy used these oral agents.

Our study reports the efficacy of G-based regimens, alone or in combination with other agents. The RR, DCR, PFS, and OS of G-based regimens, alone or in combination with other agents were 16.7%, 52.8%, 4.0 months and 7.8 months. Previously, G alone in various dosages and schedules or in combination with P, F, or other agents have shown considerable efficacy up to 60% of RR, 90% or more of DCR, and from 5.0 to 16.0 months of the median OS in BTC [11,25-27]. G-based chemotherapy in our study showed a relatively low efficacy compared with the efficacy reported in some previous studies [25-27]. One of possible explanation for this difference is that patients treated with G-based regimens in our study had a worse PS compared with patients in other studies, especially prospective studies [28,29]. Moreover, the G-based group showed a trend for a worse PS compared with the F-based group (P = 0.06). It looks unavoidable due to the retrospective nature of the present study. In one phase II study and another retrospective study which reported the effect of G-based chemotherapy, only 0% and 5% of patients had ECOG PS ≥ 2, while 16% of patients in this study had ECOG PS ≥ 2 [29,30].

Although F and G are two active chemotherapeutic agents in BTC, a large population-based randomized study which directly compares F-based regimens with G-based regimens has not been performed. Our results, through direct comparison of F-based with G-based regimens, suggest similar efficacy in terms of RR, DCR, median PFS, and OS in both chemotherapeutic agents.

Platinum compounds, such as cisplatin and oxaliplatin, have been tested in single or combination regimens in BTC. Cisplatin monotherapy has shown mostly unsatisfactory activity [31], while oxaliplatin was reported to have single-agent activity in BTC [17]. Furthermore, P was reported to have synergistic effects with G or F in preclinical studies [32,33], but the benefit on addition of P to G or F has not been clarified due to a lack of large-scaled phase III trials. Our study showed a possible benefit on the addition of P to G or F, even if it had a minimal benefit only in terms of disease control. As our non-P group was older and had a poorer PS than our P group, we also analyzed the efficacy for patients less than 65 years. This subgroup analysis only showed a minimal benefit in terms of disease control as in total patients.

In our study, 83 (52%) of 144 patients who received F-based chemotherapy received FP, while 94 (95%) of 99 patients who received G-based chemotherapy received GP (P < 0.0001). Thus comparison of F-based with G-based chemotherapy in fact means comparison of F-based with GP in the present study. However, FP and GP showed similar efficacy in subgroup analysis performed for more detailed explanation.

Recently, a pooled analysis of clinical trials including 104 trials was conducted [34]. A total of 2810 patients with BTC were analyzed. Subgroup analysis showed a significantly higher RR for gallbladder carcinoma (GBC) compared with cholangiocarcinoma (CC), but longer OS for cholangiocarcinoma, and GP was suggested as the most active regimen in BTC. Another subgroup analysis in this pooled analysis compared patients treated with regimens containing a specific drug (F, G, and P, etc.) with patients treated with regimens not containing this drug. Treatment with G and P-containing regimens demonstrated consistently higher RR and DCR compared with G-free as well as P-free regimens. In contrast, our study didn't show a difference of treatment efficacy between GBC and non-GBC in BTC, and also the efficacy of GP was similar to that of FP. As comparison of F *vs*. F-free regimens means F *vs*. G in our study, it's not feasible to perform subgroup analysis like that of the previous study. In a recent retrospective study of survival benefits of palliative chemotherapy for unresectable BTC, 179 patients treated with palliative chemotherapy were compared with 125 patients treated with the best supportive care (BSC) [30]. This study showed that the chemotherapy group had a longer survival time than the BSC group. In addition, G was more effective than 5-FU-based or cisplatin-based regimens, with a reduction in mortality of about 50%. Those results are contrary to our results, which suggest that G-based and F-based regimens have similar efficacy. However, it is hard to compare directly the efficacy of regimens because the classifications of chemotherapy regimens in both studies were different.

Studies on newer targeted agents, such as erlotinib and cetuximab, are increasing in BTC, as in other malignancies [35,36]. However, because the efficacy of these drugs is still investigational, F-based or G-based chemotherapy may be backbone of palliative chemotherapy in unresectable BTC for some time.

Our study has several limitations. Most of all, although baseline characteristics of patients were statistically similar between chemotherapy groups, our study is a nonrandomized and retrospective study. Consequently, unexpected bias may exist. In addition, F used in this study included various agents such as 5-FU, UFT, capecitabine, and S-1. Thus, the efficacy of each drug in F can be different from that of total F-based chemotherapy in this study. If we analyze independently each drug in F, however, it will not be easy to interpret exactly the results due to a small number of patients who received each agent. The efficacy of F-based *vs*. G-based chemotherapy may seem to be similar because the number of patients included in each group was not enough to show the difference. Moreover, we excluded patients who received F and G at the same time or didn't receive F or G at all because they were inconsistent with the objective of this study. Even though a small number of patients were excluded, it will also contribute potentially to selection bias in our study.

However, our study has significance in spite of these several limitations. BTC are rare tumors, but our study is a relatively large population-based study due to higher incidence of BTC in Korea. Moreover, the patients in this study received similar supportive care in a single center during chemotherapy. Further randomized controlled trials are needed in BTC.

## Conclusion

In conclusion, our results suggest that F-based and G-based chemotherapy show similar efficacy in terms of RR, DCR, PFS, and OS in BTC. In addition, the beneficial effect of adding platinum to G or F was not significant except in terms of DCR. Further prospective studies to define the efficacy of various chemotherapeutic regimens in BTC are warranted.

## Competing interests

The authors declare that they have no competing interests.

## Authors' contributions

MJK carried out this study, and drafted this manuscript. DYO conceived the design of this study, and participated in coordination of the present study. SHL and DWK performed the statistical analysis of the study. SAI and TYK participated in patient care and coordination. DSH helped to draft the manuscript and to interpret the results. YJB participated in the coordination of this study and instructed the collaborators of this manuscript. All authors read and approved the final manuscript.

## Pre-publication history

The pre-publication history for this paper can be accessed here:


